# Massive Septic Shock and Complex Survivorship in a Medically Complex Pediatric Patient Following Gastrostomy Tube Malposition With Operative Findings of Feed Within the Retrorectus Space

**DOI:** 10.7759/cureus.106785

**Published:** 2026-04-10

**Authors:** Samira Haberman, Christopher M Ahmad, Olivia Kaufman, Rae-Anne Kastle, Brett Dunbar

**Affiliations:** 1 Medical School, Kansas City University of Medicine and Biosciences, Joplin, USA; 2 General Surgery, Mercy Hospital Pittsburg, Pittsburg, USA

**Keywords:** caregiver burden, gastrostomy tube complications, necrotizing fasciitis, neurodevelopmental disability, pediatric atrial fibrillation, pediatric critical care, pediatric septic shock, post-intensive care syndrome, retrorectus abscess, sepsis survivorship

## Abstract

Gastrostomy tube (G-tube) replacement is often routine; however, in children with complex medical needs, misplacement can rapidly lead to life-threatening complications. We describe the case of a 16-year-old male patient with neuronal ceroid lipofuscinosis who developed fulminant septic shock after accidental G-tube dislodgement and malposition. At an outside hospital, reinsertion attempts required tract dilation; bloody aspirate and inconclusive radiographs were overlooked, and feeds were resumed at home. After 48 hours, the patient presented to a community hospital in profound shock (blood pressure 40/20 mmHg, lactate 7.8 mmol/L, procalcitonin >50 ng/mL) with extensive abdominal wall emphysema and feed extravasation into the scrotum. Despite maximal fluid resuscitation, he required norepinephrine, epinephrine, vasopressin, broad-spectrum antibiotics, intubation, and central line placement. Pediatric surgery at a tertiary center advised immediate source control prior to transfer; local surgeons performed emergent laparotomy, washout, and vacuum-assisted closure. Cultures grew *Klebsiella pneumoniae*, *Enterococcus faecalis*, and *Bacillus cereus*. His pediatric intensive care unit (PICU) course included necrotizing soft tissue infection of the abdominal wall, atrial fibrillation requiring cardioversion, repeated debridements, and two skin grafts. Discharged after 91 days, he required ongoing wound care and experienced multiple readmissions for pneumonia and septic episodes. This case illustrates how routine G-tube replacement can escalate into catastrophic sepsis in patients with complex medical needs. Early recognition, confirmatory imaging, prompt source control, and aggressive multidisciplinary care were lifesaving. Survivorship challenges, including repeated infections, wound care, and caregiver burden, underscore the importance of structured post-sepsis planning.

## Introduction

Medically complex children with neurodevelopmental disabilities frequently require gastrostomy tubes (G-tubes) for long-term nutritional support, placing them at a higher risk for device-related complications [1]. This risk is possibly amplified in nonverbal patients, in whom early signs of G-tube malfunction, such as discomfort, feeding intolerance, or localized pain, may be subtle, atypical, or entirely unrecognized [2]. Delayed symptom recognition in this population represents a systemic vulnerability that can allow minor mechanical issues to progress to life-threatening complications.

Children with underlying neurologic disease comprise a substantial proportion of those requiring G-tube placement. In a large retrospective study, Naiditch et al. found that 37.1% of children undergoing G-tube placement between 2006 and 2009 had an underlying neurologic condition, and tube malfunction was a prevalent complication [3]. Of the children with a neurologic condition, tube malposition occurred in 1.3% during hospitalization, which sharply rose to 27.7% following discharge, highlighting a marked shift in risk from inpatient to outpatient settings. During the same study period, G-tube dislodgement alone accounted for 50 emergency department visits, underscoring the downstream burden on acute care services [3]. 

G-tube replacement is considered a low-risk procedure, as the tubes are placed under radiographic guidance without general anesthesia [4]; however, malposition can have serious consequences. Evaluation of suspected G-tube malposition may be challenging when initial radiographs are nondiagnostic and bedside flushing is used as a surrogate for confirmation of intragastric placement. Improper tube placement or dislodgement may lead to enteral feed extravasation, superficial and deep soft tissue infection, and progression to septic shock [5]. In rare cases, enteral feeds may extravasate into the retrorectus space, a potential space between the rectus abdominis muscle and posterior rectus sheath, where leakage may be difficult to recognize early [6]. Otherwise, the literature is limited on the rates of infection in tube feeds other than percutaneous endoscopic gastrostomy (PEG) tubes. In this context, G-tube-related complications represent an important and potentially preventable source of iatrogenic, device-associated sepsis in a high-risk pediatric patient.

Although G-tube placement and replacement are common in pediatric care, particularly among children with neurologic impairment and long-term feeding dependence, clinically significant infectious complications remain relatively uncommon compared with more frequent mechanical issues such as leakage, dislodgement, and malposition. When infection does occur, it may be polymicrobial and may involve enteric organisms or skin-associated flora, depending on the depth and extent of contamination. In cases of suspected abdominal wall contamination, deep soft tissue infection, or septic shock, early broad-spectrum antimicrobial therapy is warranted while imaging, operative findings, and culture data clarify the source and microbiology [6-9].

Here, we report a case that is notable for the convergence of a rare anatomic complication, retrorectus enteral feed extravasation, with fulminant septic shock in a nonverbal child with neuronal ceroid lipofuscinosis, followed by a prolonged and medically burdensome recovery. Diagnostic delay related to nondiagnostic imaging and bedside confirmation of tube position highlights a systems-level vulnerability in the evaluation of suspected G-tube malfunction. Beyond survival, this case underscores the underrecognized long-term consequences of pediatric sepsis in children with complex chronic conditions, including prolonged recovery and family impact, which remain underreported in the literature [10]. The purpose of this report is to emphasize the risk of delayed recognition of G-tube malposition, illustrate a rare but catastrophic complication, and highlight the importance of multidisciplinary care and long-term support for pediatric sepsis survivors and their families.

## Case presentation

**Figure 1:**
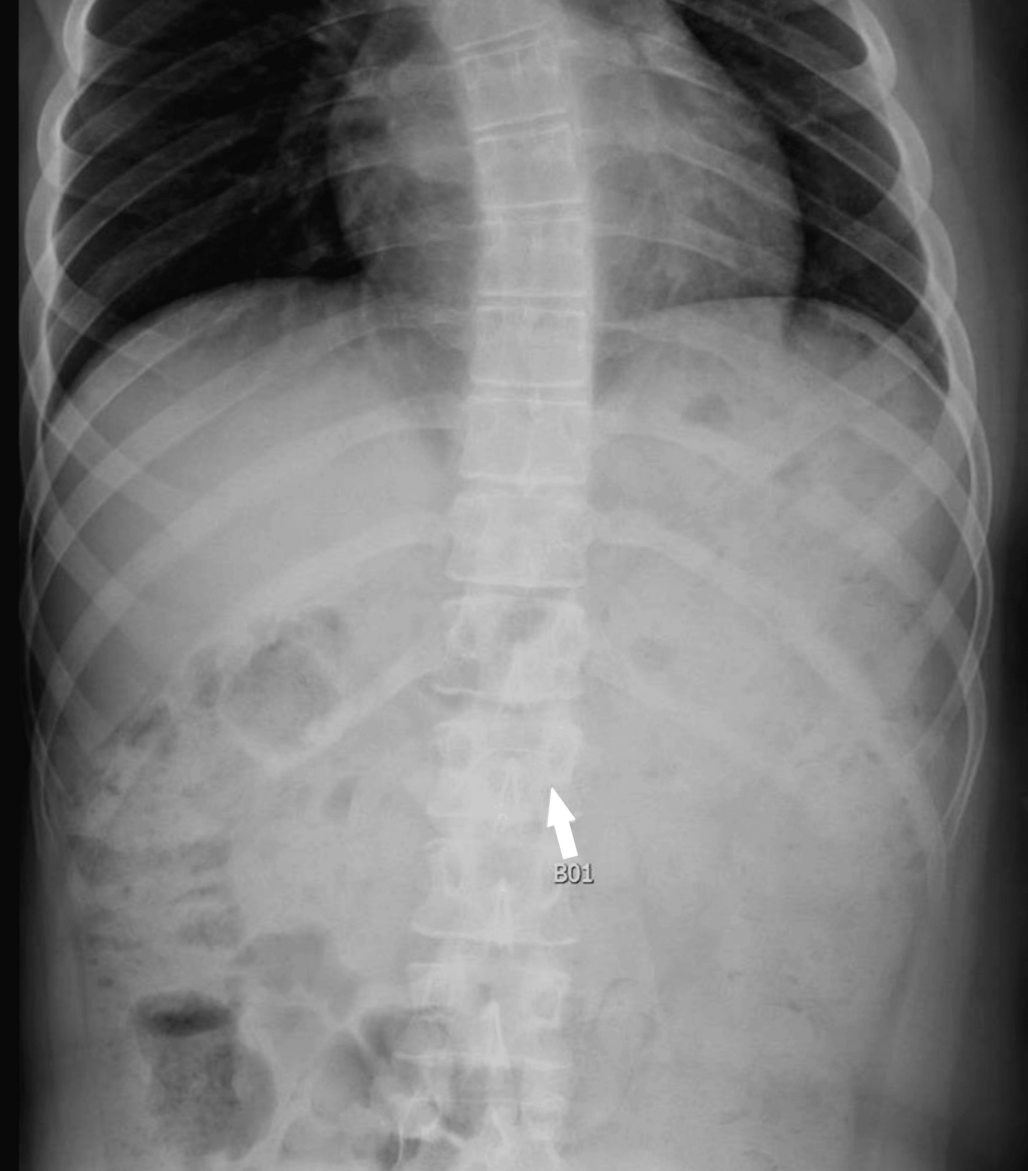
Initial abdominal radiograph obtained to evaluate gastrostomy tube placement. Anteroposterior and lateral views demonstrating the gastrostomy tube in situ but without clear visualization of the distal tip within the gastric lumen. The white arrow indicates a vertebral body used as an anatomic reference point, emphasizing that the gastrostomy tube tip is not definitively identified on the radiograph, rendering the imaging nondiagnostic.

A 16-year-old male patient with neuronal ceroid lipofuscinosis, complicated by severe developmental regression, blindness, epilepsy, and G-tube dependence, accidentally dislodged his G-tube with the retention balloon still inflated. Caregivers and a home healthcare supervisor attempted bedside replacement without success, prompting evaluation at a local emergency department. The G-tube was replaced after multiple attempts using serial dilators. Tube position was assessed by review of abdominal radiographs and by flushing air and tap water through the tube. During reinsertion, bloody aspirate was noted during flushing and attributed to tract trauma. An initial abdominal radiograph was obtained during tube replacement evaluation, but the distal tube tip could not be definitively confirmed within the gastric lumen, as shown in Figure 1. 

Despite caregiver concerns regarding tube positioning, the patient was discharged with reassurance that the tube was appropriately placed. Return precautions were provided. 

Over the subsequent two days, the patient developed progressive fever, lethargy, pallor, and hypoxia. Caregivers noted that overnight tube feeds were infused more slowly than usual. Due to worsening clinical status, emergency medical services (EMS) were activated.

Upon EMS arrival, the patient was minimally responsive with profound hypotension (blood pressure 40/20 mmHg) and hypoxia (peripheral oxygen saturation (SpO₂) 80%) in the emergency department. Initial vital signs included temperature 37.4°C, heart rate 125 beats per minute, respiratory rate 32 breaths per minute, blood pressure 69/34 mmHg after administration of 3 liters of normal saline, SpO₂ 90% on 15 L/minute via nonrebreather mask, and weight 82 kg. Physical examination demonstrated severe distress with marked abdominal distension, mottling, and ecchymosis over the left flank, and cold extremities. Imaging and operative exploration confirmed that the G-tube was malpositioned in the retrorectus space rather than the gastric lumen.

Initial venous blood gas revealed a pH 7.31, partial pressure of carbon dioxide (pCO₂) 41 mmHg, bicarbonate 20 mmol/L, base deficit −6, and lactate 7.8 mmol/L. Laboratory evaluation demonstrated acute kidney injury (creatinine 2.7 mg/dL), transaminitis (aspartate aminotransferase (AST) and alanine transaminase (ALT) in the 160s U/L), C-reactive protein (CRP) 31.3 mg/dL, and procalcitonin >50 ng/mL. The patient received an additional 3 liters of normal saline, was started on vancomycin and piperacillin-tazobactam, and initiated on norepinephrine for refractory shock. Initial laboratory findings on presentation are summarized in Table 1.

**Table 1:** Admission laboratory values at presentation with corresponding reference ranges and clinical interpretation ALT, alanine aminotransferase; AST, aspartate aminotransferase; CRP, C-reactive protein

Parameter	Patient Value	Reference Range	Clinical Interpretation
Lactate	7.8 mmol/L	0.5–2.0 mmol/L	Severe lactic acidosis consistent with septic shock
Creatinine	2.7 mg/dL	0.5–1.0 mg/dL	Acute kidney injury
AST	162 U/L	10–40 U/L	Hepatic injury (transaminitis)
ALT	146 U/L	7–56 U/L	Hepatic injury (transaminitis)
CRP	31.3 mg/dL	<0.5 mg/dL	Marked systemic inflammation
Procalcitonin	>50 ng/mL	<0.1 ng/mL	Severe bacterial infection / septic shock
Platelets	115 × 10³/µL	150–450 × 10³/µL	Thrombocytopenia, possible consumptive process
Venous pH	7.31	7.31–7.41	Borderline acidemia
Base deficit	−6	−2 to +2	Metabolic acidosis

**Figure 2:**
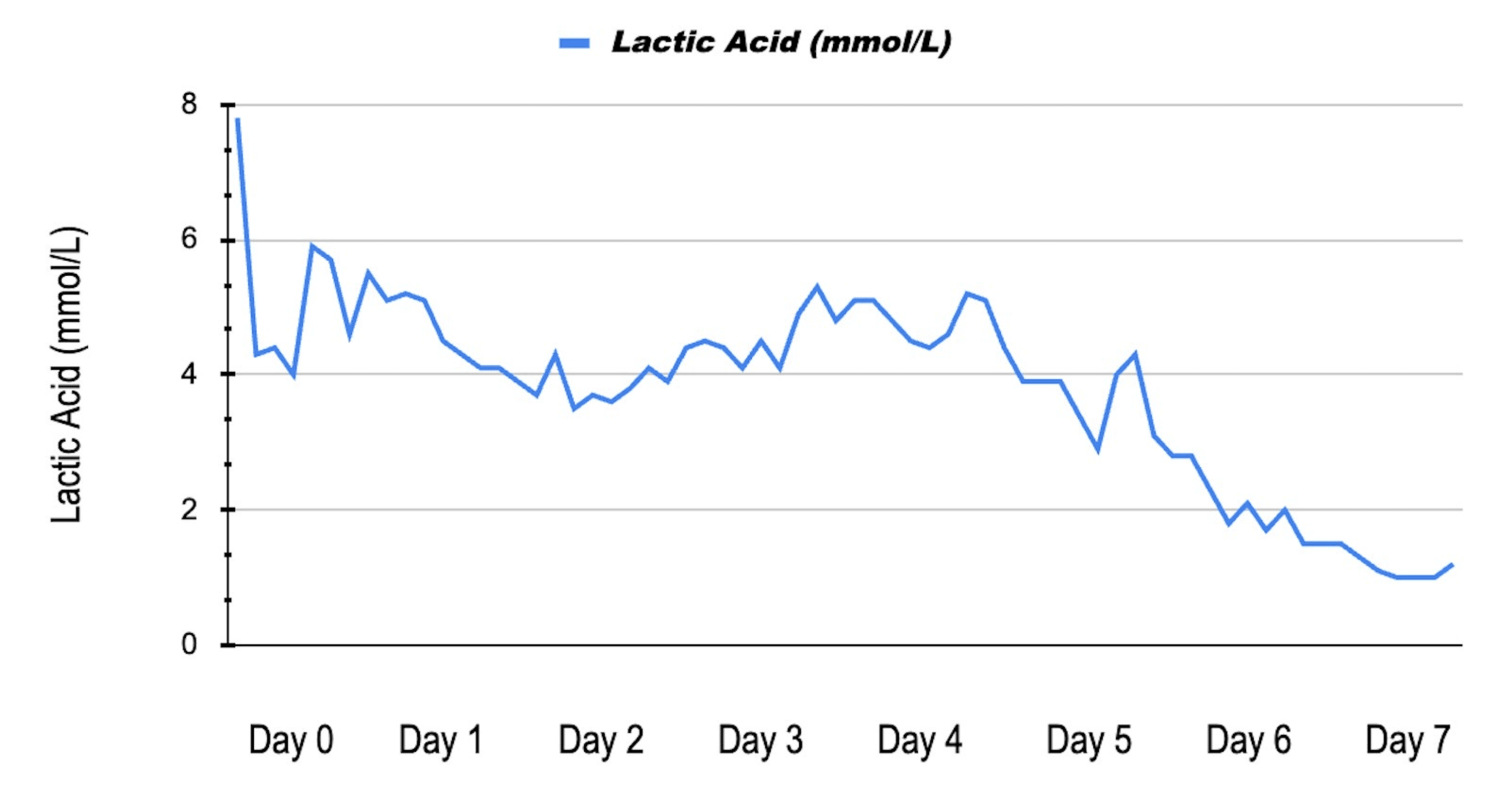
Trend of serum lactic acid (mmol/L) from Day 0 to Day 7. The graph demonstrates a peak of 7.8 mmol/L on admission (Day 0), followed by a progressive downward trend toward normalization (normalization = <2.0 mmol/L ) following emergent surgical washout and hemodynamic stabilization.

Serial serum lactate values demonstrated gradual improvement after operative source control and hemodynamic stabilization, as shown in Figure 2.

**Figure 3:**
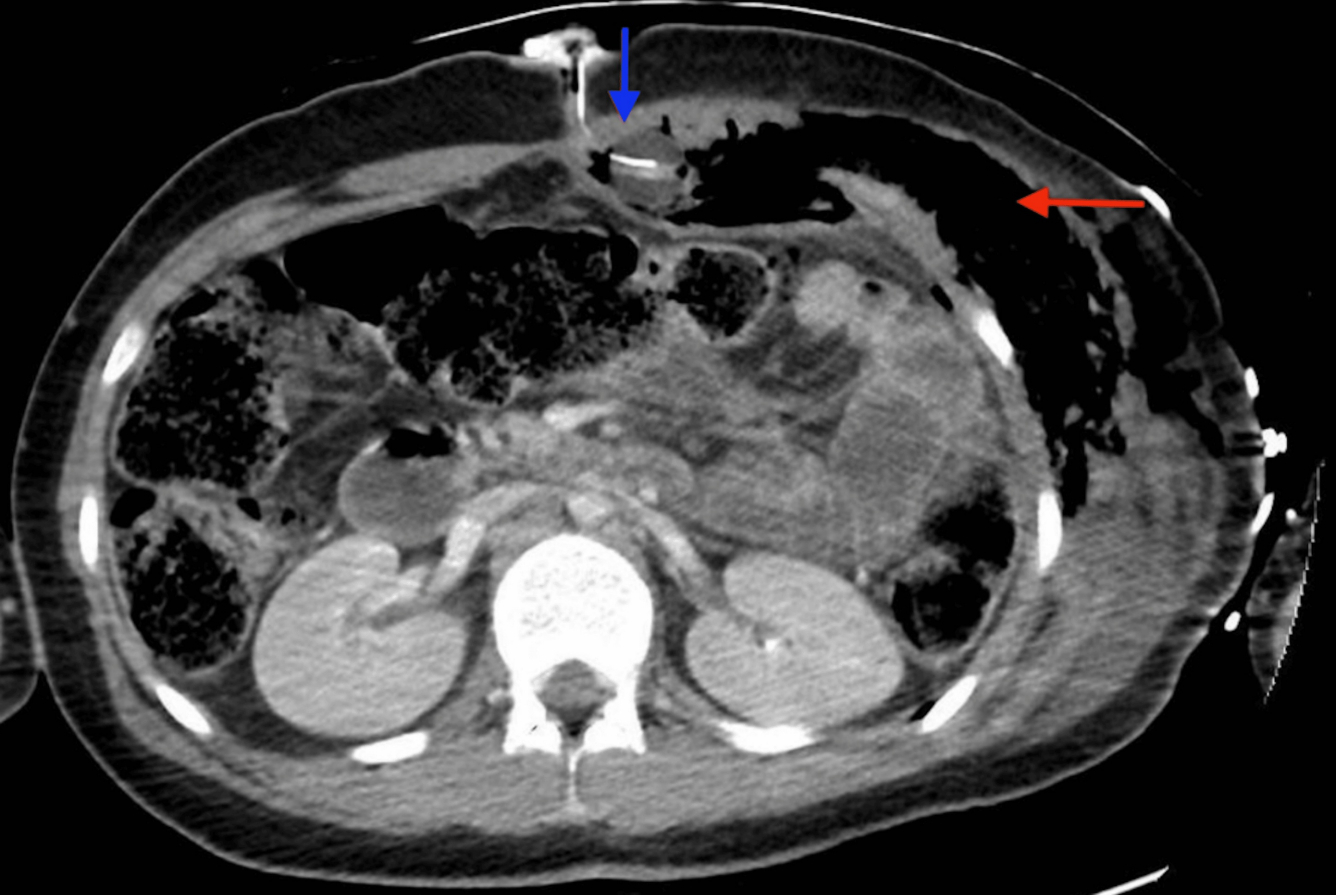
Axial contrast-enhanced CT of the abdomen demonstrating a malpositioned gastrostomy tube. The tube tip is located within the retrorectus space (blue arrow), with associated extraluminal enteral contents tracking within the anterior abdominal wall (red arrow), consistent with gastrostomy tube displacement and leakage.

Computed tomography (CT) demonstrated a malpositioned G-tube with the tip residing in the retrorectus space and extensive extravasation of enteral contents into the abdominal wall (Figure 3).

**Figure 4:**
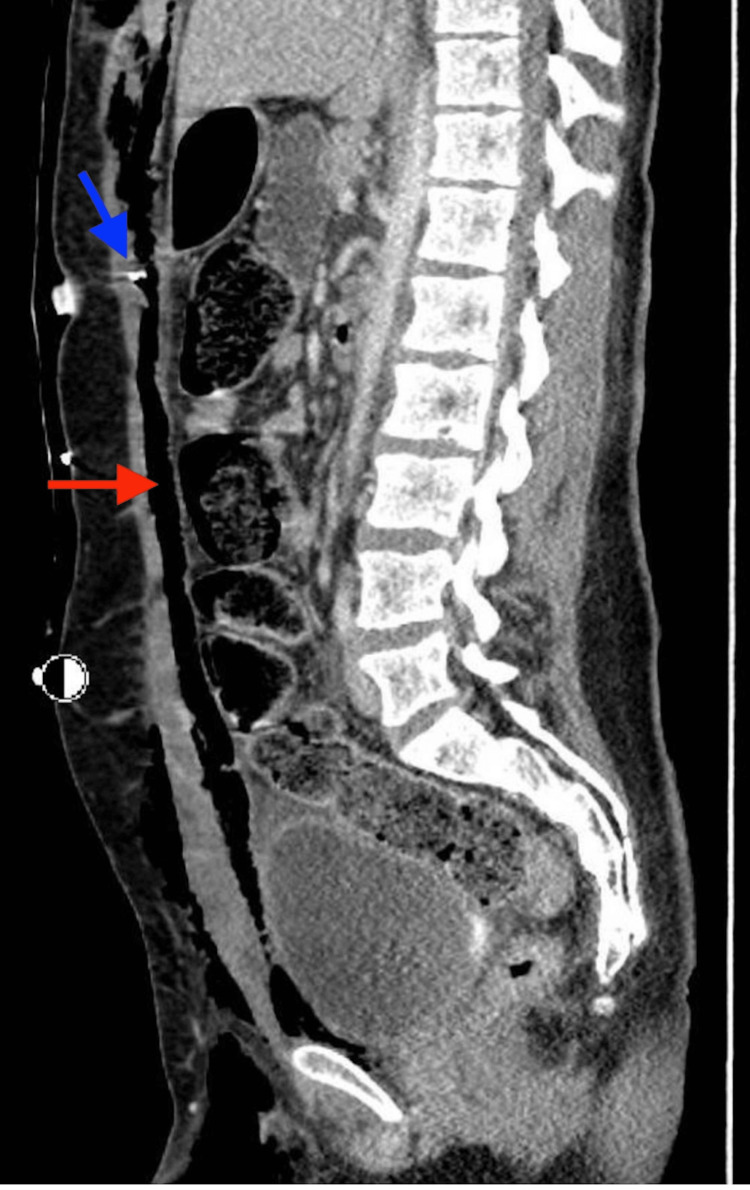
Sagittal CT of the chest, abdomen, and pelvis with intravenous contrast. Vertical tracking of extraluminal air and enteric contents anterior to the peritoneum from the malpositioned gastrostomy tube site. The blue arrow indicates the malpositioned gastrostomy tube in the retrorectus space. The red arrow indicates the extraluminal air and enteric contents anterior to the peritoneum.

Sagittal CT reconstruction further demonstrated the vertical extent of extraluminal air and enteric contents anterior to the peritoneum, as shown in Figure 4.

The outside emergency department consulted pediatric intensive care and pediatric surgery at a tertiary center. Due to a lack of air transport availability secondary to weather conditions and the unavailability of ground EMS transport resources, the receiving teams recommended emergent exploratory laparotomy at the presenting hospital and initiation of an epinephrine infusion.

The patient deteriorated with escalating vasopressor requirements, underwent endotracheal intubation and subclavian central venous catheter placement, and was taken emergently to the operating room for exploratory laparotomy and abdominal washout. Operative findings revealed a large volume of tube feeds within the retrorectus space. The abdomen was left open. Wound cultures grew moderate *Klebsiella pneumoniae* and heavy *Enterococcus faecalis*. Blood cultures grew *K. pneumoniae* at 13 hours. Microbiology results are summarized in Table 2.

**Table 2:** Microbiology results, susceptibility findings, and clinical relevance

Source	Organisms Identified	Key Susceptibility/Resistance Findings	Clinical Relevance
Initial wound cultures	Klebsiella pneumoniae, Enterococcus faecalis	*Klebsiella* resistant to ampicillin and ampicillin-sulbactam; *E. faecalis* susceptible to ampicillin	Supported broad abdominal/soft tissue coverage
Blood cultures	Klebsiella pneumoniae	Blood culture isolate was consistent with *K. pneumoniae* bacteremia; full susceptibility details were not separately reported.	Confirmed bloodstream dissemination
Subsequent wound cultures	*Candida* species	Antifungal susceptibility not routinely reported/not available	Supported prolonged antifungal therapy
Later abscess	*Pseudomonas* species	Susceptibility profile per hospital microbiology report	Guided later targeted therapy

Postoperatively, the patient was transferred to the pediatric intensive care unit (PICU). On arrival, vital signs included temperature 37.4°C, heart rate 125 beats per minute, blood pressure 90/48 mmHg, and oxygen saturation 96% on a fraction of inspired oxygen (FiO₂) 1.0. He was receiving norepinephrine at 1.5 µg/kg/minute, epinephrine at 0.41 µg/kg/minute, and vasopressin at 1 milliunit/kg/minute.

Initial PICU laboratory studies demonstrated blood urea nitrogen 34 mg/dL, creatinine 1.87 mg/dL, AST 266 U/L, ALT 123 U/L, albumin 2.2 g/dL, CRP 28.2 mg/dL, ferritin 382 ng/mL, white blood cell count 1.4 × 10³/µL, hemoglobin 12.9 g/dL, hematocrit 39%, platelets 95 × 10³/µL, international normalized ratio (INR) 1.5, prothrombin time 15 seconds, partial thromboplastin time 38 seconds, and fibrinogen 538 mg/dL. Arterial blood gas showed pH 7.17, pCO₂ 40 mmHg, pO₂ 145 mmHg, bicarbonate 14 mmol/L, base deficit −14, and lactate 4.3 mmol/L. Transthoracic echocardiography demonstrated a left ventricular ejection fraction (LVEF) of 36% with mild mitral regurgitation.

He was placed on extracorporeal membrane oxygenation (ECMO) watch and treated with calcium chloride infusion and stress-dose corticosteroids. Antimicrobial therapy was broadened to include cefepime, metronidazole, vancomycin, caspofungin, and clindamycin. He returned to the operating room with pediatric surgery and urology, where residual purulent fluid was identified in the retrorectus space. A Penrose drain was placed in the left lower quadrant and the flank subcutaneous fluid collection. Air was noted within the scrotum without purulent material or evidence of necrotizing soft tissue infection. Necrosis and friability of the inferior omentum were observed and resected. Negative-pressure abdominal therapy (AbThera™; 3M company, Maplewood, Minnesota, United States) was replaced.

From Hospital Day 1 through 7, the patient experienced persistent hemodynamic instability with difficulty weaning vasopressors. He remained on norepinephrine, epinephrine, vasopressin, and calcium chloride infusion, with a net positive fluid balance of approximately 6 liters during the first 40 hours. Methylene blue (2 mg/kg IV) was administered on days 1 and 2. Repeat echocardiography on Day 4 demonstrated an LVEF of 29% with mild tricuspid and mitral regurgitation and left atrial dilation; milrinone was initiated. By Day 10, LVEF improved to 63%, and milrinone was discontinued. Echocardiographic findings and vasopressor requirements are summarized in Table 3.

**Table 3:** Ventilation and respiratory milestones This table of ventilation and respiratory milestones shows the temporal correlation between vasopressor use and myocardial recovery Epi, epinephrine; LVEF, left ventricular ejection fraction; MR, mitral regurgitation; NE, norepinephrine; TR, tricuspid regurgitation; ↑, dose increase

Hospital Day	Vasopressors/Inotropes	Echocardiographic Findings
Day 0: Septic shock presentation	NE, Epi, Vasopressin	LVEF 36%, mild MR
Day 1	NE ↑, Epi ↑, Vasopressin	Persistent myocardial dysfunction
Day 2	NE, Epi, Vasopressin, Milrinone	LVEF 29%, mild TR/MR
Day 4	Weaning vasopressors	LVEF improved to 52%
Day 7	Off Milrinone	LVEF 63%

Microbiologic data from outside-hospital cultures demonstrated *K. pneumoniae* resistant to ampicillin and ampicillin-sulbactam, and *E. faecalis* susceptible to ampicillin, along with moderate growth of presumptive coagulase-positive *Staphylococcus*. Source control required multiple operative debridements on days 3, 4, and 5, with burn surgery and plastic surgery consulted intraoperatively for debridement planning and AbThera management. A summary of surgical interventions and operative findings is provided in Table 4.

**Table 4:** Summary of surgical interventions and operative findings CT, computed tomography; Wound vac, vacuum-assisted closure

Hospital Day	Intervention	Key Findings
Day 0: Septic shock presentation	Exploratory laparotomy	Large-volume feed extravasation in retrorectus space
Day 1	Re-exploration/debridement	Residual purulence; necrotic omentum
Day 3	Debridement	Progressive soft tissue necrosis
Day 4	Debridement	Burn/plastics consulted intraoperatively
Day 5	Debridement	Continued source control
Day 7	Abdominal closure	Wound vac placement
Day 54	CT-guided drainage	Pseudomonas abscess anterior to bladder
Day 62	Skin grafting	Definitive wound coverage

**Figure 5:**
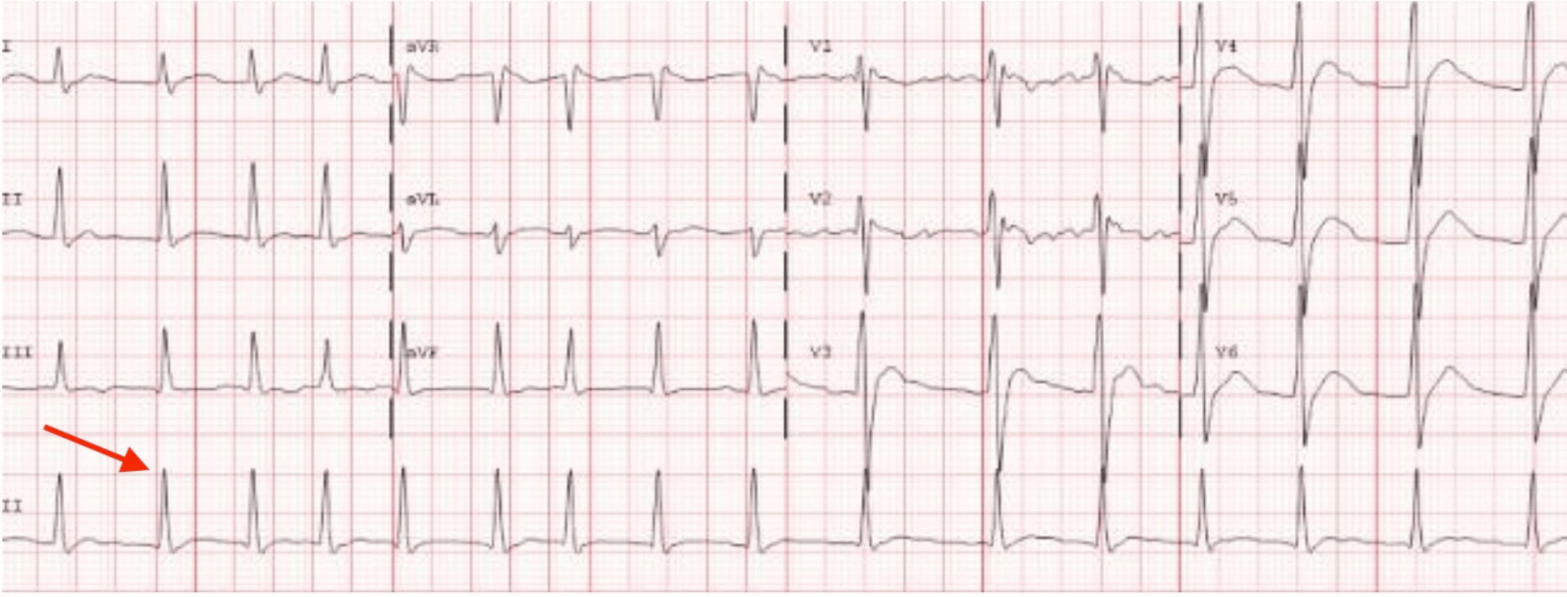
Electrocardiographic evidence of new-onset atrial fibrillation (red arrow) The tracing illustrates an episode of atrial fibrillation with rapid ventricular response occurring during the period of maximal physiologic stress. This event required emergent synchronized cardioversion due to associated hemodynamic instability, resulting in a successful return to sinus rhythm.

The hospital course was complicated by atrial fibrillation requiring cardioversion, respiratory failure necessitating reintubation, and hospital-acquired pneumonia. An electrocardiogram demonstrating atrial fibrillation is shown in Figure 5.

Additional events included abdominal closure with wound vacuum therapy on Day 7, *Candida*-positive wound cultures prompting prolonged caspofungin therapy (32 days), extubation to high-flow nasal cannula on Day 20, reintubation on Day 23, and subsequent extubation to bilevel positive airway pressure on Day 32. The patient was transferred to the general pediatric floor on Day 44 on a low-flow nasal cannula.

On Day 54, he underwent CT-guided drainage of a newly identified abscess anterior to the bladder that grew *Pseudomonas*. He subsequently underwent split-thickness skin grafting on Day 62. He returned to the PICU on Day 67 for pneumonia treated with piperacillin-tazobactam, was transferred back to the floor on Day 73, completed antibiotic therapy on Day 88, and was discharged home on hospital Day 91.

Following discharge, the patient required home health nursing support and longitudinal follow-up with primary care and subspecialties, including pulmonology, cardiology, gastroenterology, neurology, and nephrology. Post-discharge care was complicated by the patient’s underlying neuronal ceroid lipofuscinosis, which significantly impaired wound healing and rehabilitation. Caregivers reported recurrent readmissions associated with significant caregiver distress.

Subsequent hospitalizations occurred at approximately 10 months post-discharge for COVID-19-associated septic shock with respiratory failure (Days 191-193), at 12 months for *Pseudomonas* septic shock with respiratory failure (Days 262-289), at 14 months for *Pseudomonas *community-acquired pneumonia with respiratory failure (Days 338-341), and at 15 months for culture-negative community-acquired pneumonia with respiratory failure (Days 354-361). Similar patterns continued into the following years, with *Staphylococcus aureus* septic shock and respiratory failure beginning at Month 19, and again in Month 29. Caregivers and clinicians noted progressive decline consistent with the patient’s underlying neurodegenerative disease, with continued deterioration in neurologic baseline and daily functioning. A detailed chronology of the patient’s clinical course, including diagnostic findings, operative interventions, and key management milestones, is summarized in Table 5.

**Table 5:** Clinical timeline and management AKI, acute kidney injury; BiPAP, bilevel positive airway pressure; BP, blood pressure; CT, computed tomography; ED, emergency department; EMS, emergency medical services; G-tube, gastrostomy tube; LVEF, left ventricular ejection fraction; PICU, pediatric intensive care unit.

Hospital Day	Event
48 hours prior to shock presentation	Accidental G-tube dislodgement at home with balloon intact
48 hours prior to shock presentation	Evaluation at local ED; G-tube replaced after multiple attempts and tract dilation
48 hours prior to shock presentation	Bloody aspirate noted during flushing; abdominal radiographs inconclusive
48 hours prior to shock presentation	Discharged home with return precautions
—	Progressive fever, lethargy, pallor, hypoxia; slow feed infusion at home
Day 0: Septic shock presentation	EMS activation for hypotension (BP 40/20 mmHg) and hypoxia
Day 0: Septic shock presentation	Community ED: septic shock, lactate 7.8 mmol/L, AKI, transaminitis
Day 0: Septic shock presentation	CT chest/abdomen/pelvis shows G-tube malposition and extraluminal air
Day 0: Septic shock presentation	Emergent exploratory laparotomy and washout at community hospital
Day 0: Septic shock presentation	Retrorectus feed extravasation identified; abdomen left open
Day 0: Septic shock presentation	Transfer to tertiary PICU post-operatively
Days 1–5	Persistent shock; serial operative debridements
Days 1–2	Methylene blue administered for refractory vasoplegia
Day 2	Echocardiogram: LVEF 29%; milrinone initiated
Day 4	Echocardiogram: LVEF improved to 52%
Day 7	Abdominal closure; wound vac placement
Day 20	Extubated to high-flow nasal cannula
Day 23	Re-intubated for respiratory failure
Day 32	Extubated to BiPAP
Day 54	CT-guided drainage of Pseudomonas abscess
Day 62	Split-thickness skin graft
Day 91	Discharged home

## Discussion

This case demonstrates rapid progression from a seemingly routine procedure to catastrophic septic shock in a neurodegenerative pediatric patient. G-tube malposition during reinsertion resulted in feed extravasation into the abdominal wall and retrorectus space, with extensive soft-tissue emphysema tracking into the scrotum and chest, complicated by necrotizing soft-tissue infection and polymicrobial sepsis. Early indicators, including bloody aspirate during flushing and nondiagnostic initial radiographs, were present prior to clinical deterioration, emphasizing the importance of careful confirmation when tube position is uncertain [5,7]. In retrospect, the combination of difficult reinsertion, bloody aspirate during flushing, nondiagnostic radiographs, slowed feed infusion, progressive abdominal distension, and early systemic decline represented a high-risk diagnostic pattern that should prompt urgent reassessment of tube position and possible extraluminal placement.

The acute course underscores core sepsis management principles: early broad-spectrum antibiotics, aggressive hemodynamic support, and timely surgical source control [8,9]. In this case, the initial empiric use of vancomycin and piperacillin-tazobactam was selected to provide broad coverage for possible polymicrobial abdominal, soft tissue, and bloodstream infection while operative source control and culture data were pending. A key operational feature of this case was that transfer to a tertiary center was constrained by weather and transport availability, prompting the receiving PICU and pediatric surgery teams to recommend emergent exploratory laparotomy at the presenting facility, consistent with the clinical imperative to prioritize source control when instability or logistics prevent safe deferral [9].

**Figure 6:**
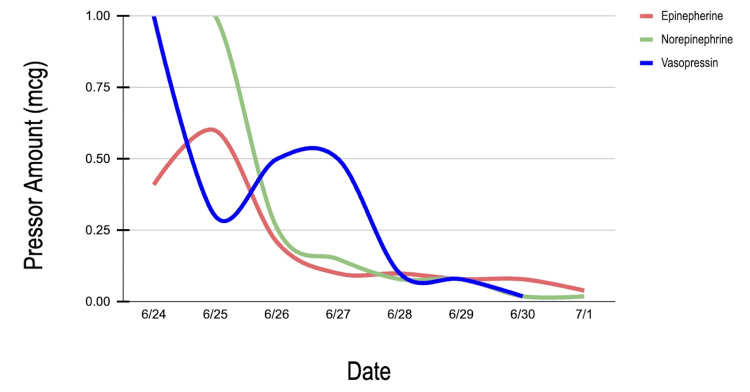
Vasoactive support requirements from hospital days 0–7 in the patient of the current report. The graph demonstrates the requirement for multiple vasopressors (epinephrine, norepinephrine, and vasopressin) during the period of refractory shock. The weaning of pressor support correlates with the stabilization of hemodynamic parameters and the improvement of lactic acidosis following operative management.

The occurrence of atrial fibrillation and decreased left ventricular ejection fraction further illustrates that sepsis can precipitate clinically significant rhythm disturbances and myocardial dysfunctions during critical illness [11,12]. The subsequent course included an open abdomen after retrorectus feed extravasation, transfer to a tertiary PICU, serial operative debridements, and persistent vasoplegia requiring high-dose vasoactive support, a phenomenon described in septic shock [13]. This clinical course is illustrated in Figure 6.

Post discharge, survivorship challenges became the dominant burden, including prolonged wound care needs, recurrent severe respiratory infections, and repeated critical care admissions. These features align with post-intensive care syndrome in pediatrics (PICS-P). This recognizes the functional outcomes and caregiver strain that may persist long after shock resolution [14]. Structured follow-up models for pediatric sepsis survivors have been described, but implementation remains inconsistent across systems [15]. A model by Manning et al. presents a framework highlighting physical, cognitive, emotional, and social health as families and patients navigate PICS-P [16].

Recurrent hospitalizations following discharge are summarized in Table 6. The unified timeline emphasizes that successful acute resuscitation does not mark the end of a critical illness; for medically complex children, the post-ICU phase can involve equal or greater complexity, requiring proactive planning, multidisciplinary follow-up, and caregiver engagement. One mishap sends the patient back into the ICU. The reality is that these children require frequent interaction with healthcare facilities throughout their childhood [10,14,15].

**Table 6:** Summary of recurrent hospitalizations and clinical presentations in the patient of the current report. CAP, community-acquired pneumonia.

Accurate Relative Day	Diagnosis	Clinical Features
Day 284	COVID-19-associated septic shock	Respiratory failure
Day 355	Pseudomonas septic shock	Respiratory failure
Day 431	Pseudomonas CAP	Respiratory failure
Day 447	Culture-negative CAP	Respiratory failure

Clinically, this case reinforces that when G-tube reinsertion is difficult or radiographic confirmation is equivocal, clinicians should maintain a low threshold for repeat imaging, contrast confirmation, surgical consultation, and withholding enteral feeds until intragastric placement is definitively established.

This report is limited by its single-patient design, which restricts generalizability to broader pediatric populations. The patient’s underlying neurodegenerative disorder and medical complexity likely amplified both the severity of septic shock and the prolonged recovery course, and outcomes may differ in otherwise healthy children. Additionally, elements of the initial G-tube reinsertion and imaging interpretation occurred at an outside facility, and complete procedural documentation was not available for review. As a retrospective case report, causal relationships cannot be definitively established, and standardized measures of long-term post-intensive care syndrome were not formally applied. Despite these limitations, the detailed clinical timeline and operative findings provide valuable insight into a rare and catastrophic complication of G-tube malposition.

## Conclusions

This case highlights the critical importance of procedural vigilance and early recognition of evolving sepsis following G-tube replacement. Management must extend beyond acute stabilization to include comprehensive long-term recovery support. For patients with underlying neurodegenerative disorders, recognizing survivorship as an essential component of care is vital to addressing the multifaceted challenges faced by both the patient and their caregivers.
